# Prediction of risk factors for linezolid-induced thrombocytopenia based on neural network model

**DOI:** 10.3389/fphar.2024.1292828

**Published:** 2024-02-21

**Authors:** Xian Zhao, Qin Peng, Dongmei Hu, Weiwei Li, Qing Ji, Qianqian Dong, Luguang Huang, Miyang Piao, Yi Ding, Jingwen Wang

**Affiliations:** ^1^ Department of Pharmacy, First Affiliated Hospital of Air Force Medical University, Xi’an, Shaanxi, China; ^2^ Department of Hepatobiliary Surgery, First Affiliated Hospital of Air Force Medical University, Xi’an, Shaanxi, China; ^3^ Department of Information, First Affiliated Hospital of Air Force Medical University, Xi’an, Shaanxi, China

**Keywords:** ANN, logistic regression, linezolid, thrombocytopenia, risk factor, ADR

## Abstract

**Background:** Based on real-world medical data, the artificial neural network model was used to predict the risk factors of linezolid-induced thrombocytopenia to provide a reference for better clinical use of this drug and achieve the timely prevention of adverse reactions.

**Methods:** The artificial neural network algorithm was used to construct the prediction model of the risk factors of linezolid-induced thrombocytopenia and further evaluate the effectiveness of the artificial neural network model compared with the traditional Logistic regression model.

**Results:** A total of 1,837 patients receiving linezolid treatment in a hospital in Xi ‘an, Shaanxi Province from 1 January 2011 to 1 January 2021 were recruited. According to the exclusion criteria, 1,273 cases that did not meet the requirements of the study were excluded. A total of 564 valid cases were included in the study, with 89 (15.78%) having thrombocytopenia. The prediction accuracy of the artificial neural network model was 96.32%, and the AUROC was 0.944, which was significantly higher than that of the Logistic regression model, which was 86.14%, and the AUROC was 0.796. In the artificial neural network model, urea, platelet baseline value and serum albumin were among the top three important risk factors.

**Conclusion:** The predictive performance of the artificial neural network model is better than that of the traditional Logistic regression model, and it can well predict the risk factors of linezolid-induced thrombocytopenia.

## 1 Introduction

Linezolid is a widely used oxazolidinone that penetrates cerebrospinal fluid and bone tissue and exhibits good antibacterial effects against Gram-positive bacteria, especially methicillin-resistant *Staphylococcus aureus* (MRSA) and other resistant Gram-positive bacteria (Dryden, 2011; Cazavet et al., 2020). However, during the clinical application of all drugs, a serious problem cannot be avoided, namely, adverse reactions (Edwards and Aronson, 2000; Coleman and Pontefract, 2016). Thrombocytopenia is one of the major adverse reactions of linezolid, and existing studies have shown (Bernstein et al., 2003; Hanai et al., 2016a) that the mechanism of linezolid-induced thrombocytopenia, mainly including immune-mediation, myelosuppression, inhibitory release and oxidative stress. Although this adverse drug reaction is treatable, it may cause the discontinuation of linezolid therapy and have an impact on its clinical use. Studies have shown (M et al., 2022; Cossu et al., 2014; An and Wang, 2013) that reversible myelosuppression, such as thrombocytopenia and anemia, often occurs after 4–6 weeks of continuous linezolid treatment, with an incidence of 7.4%–31.3%.

In recent years, the improvement and enhancement of computer computing power and the innovation and improvement of algorithms have greatly promoted the use of machine learning. In addition, many researchers have applied machine learning to various subdivisions, such as survival prediction of gastric cancer patients (Yazdani Charati et al., 2018), risk prediction of fetal congenital heart disease (Li et al., 2017), early outcome prediction and risk classification of cardiac arrest patients in out-of-hospital intensive care (Johnsson et al., 2020), and conducted more intensified studies. Among many machine learning algorithms, Artificial Neural Network (ANN), also called Multilayer Perceptron (MLP), is one of the important algorithms of machine learning. ANN is a typical supervised learning algorithm, the creation of which is inspired by the human brain and nervous system (Pergialiotis et al., 2018). Compared with the traditional logistic regression model, ANN can be used to find the approximate mapping between the input and output patterns of patient data and realize non-intuitive and complex nonlinear separation between various types of patients.

In the present study, we reported the risk factors of linezolid-induced thrombocytopenia in a single-center retrospective study combined with several literature (Cazavet et al., 2020; M et al., 2022; Cossu et al., 2014; Niwa et al., 2009; Ikuta et al., 2011; Takahashi et al., 2011; Chen et al., 2012; Nukui et al., 2013; Ishida et al., 2013; Natsumoto et al., 2014; Ni et al., 2014; Zhang et al., 2014; Jie, 2016; Tajima et al., 2016; Choi et al., 2019; Kaya Kılıç et al., 2019; Kim et al., 2019; Qin et al., 2022). Based on real-world medical data, an artificial neural network model was used to predict the risk factors and prediction accuracy of linezolid-induced thrombocytopenia, and compared with the traditional logistic regression model, so as to further evaluate the effectiveness of the neural network model and provide a reference for better clinical use of this drug for timely prevention of the adverse drug reactions.

## 2 Materials and methods

### 2.1 Inclusion criteria

This single-center retrospective observational study was conducted in a hospital in Xi’an, Shaanxi Province. In the present study, diagnosis and treatment data were collected from the HIS system of this hospital, and the inclusion criteria for patients were having received linezolid between 1 January 2011 and 1 January 2021. The collected clinic data were stored in a Microsoft Excel file. In order to protect the privacy of patients, the collected data were anonymous, the names and/or numbers of patients were not identified, and there was no adverse effect on patients. Since this was a retrospective study, informed consent was not required for the collection of patient care data in the present study. The study protocol was reviewed and approved by the Ethics Committee of this hospital and the study was conducted according to the World Medical Association Declaration of Helsinki (THE WORLD MEDICAL ASSOCIATION and INC, 2013).

### 2.2 Exclusion criteria

The last platelet count value before linezolid use was defined as the baseline platelet value. Exclusion criteria for patients were (Hirano et al., 2014): 1) Patients with blood system diseases (such as myelosuppression, hemophilia, etc.), immune system abnormalities (such as systemic lupus erythematosus, organ transplantation, etc.), hepatoblastoma, severe acute pancreatitis; 2) chemotherapy, hemodialysis, or combined use of antiplatelet drugs (such as clopidogrel, etc.) during treatment; 3) baseline platelet values <100 × 10^9/L or >400 × 10^9/L; 4) the total number of platelet count test points was less than 3; 5) no baseline platelet values; 6) non-standard linezolid treatment programs.

### 2.3 Criteria for determining thrombocytopenia

Taking the nadir of platelet count within 15 times after linezolid use as the basis for judging whether thrombocytopenia occurs, if it was lower than the normal value of platelet count (i.e., less than 100 × 10^9/L) and at the same time lower than 75% of the baseline value of platelets, the patient was defined as having developed thrombocytopenia (Niwa et al., 2009).

### 2.4 Data collection

Taking into account the risk factors for linezolid-induced thrombocytopenia reported in the literature (Cazavet et al., 2020; M et al., 2022; Cossu et al., 2014; Niwa et al., 2009; Ikuta et al., 2011; Takahashi et al., 2011; Chen et al., 2012; Nukui et al., 2013; Ishida et al., 2013; Natsumoto et al., 2014; Ni et al., 2014; Zhang et al., 2014; Jie, 2016; Tajima et al., 2016; Choi et al., 2019; Kaya Kılıç et al., 2019; Kim et al., 2019; Qin et al., 2022), various data in clinical diagnosis and treatment of the included study subjects were recorded and assessed, including gender, age, height, weight, BMI, route of linezolid administration, total days of administration, baseline platelet values, dynamic changes in platelet count values during treatment, TP, ALB, TBIL, DBIL, urea, history of hypertension, history of diabetes, history of malignant tumors, and combined use of unfractionated heparin. Alanine aminotransferase (ALT) and aspartate aminotransferase (AST) were selected for liver enzyme parameters, and creatinine clearance (Ccr) was used to reflect the renal function. Creatinine clearance was calculated using the Cockcroft-Gault equation (Cockcroft and Gault, 1976).

### 2.5 Data analysis

Both univariate and multivariate analyses were performed using SPSS 22.0. The analysis was performed in two steps. First, univariate analysis was carried out. Categorical variables in the chart were identified as (1), expressed as n (%), with the assumption verified using the χ2 test; continuous variables were not identified, expressed as “ ± s,” with the assumption verified adjusted *t*-test. All tests were two-sided and differences were considered statistically significant at *p* < 0.05 (Takahashi et al., 2011; Hirano et al., 2014; Zhang et al., 2014). The potential risk factors for linezolid-induced thrombocytopenia were identified (factors with *p* < 0.05).

Multivariate analysis was then performed to obtain the odds ratio (OR), 95% confidence interval (95% CI), and *p*-value of each potential risk factor to determine the independent risk factors for linezolid-induced thrombocytopenia (factors with *p* < 0.05).

### 2.6 Data processing

**FIGURE 1:**
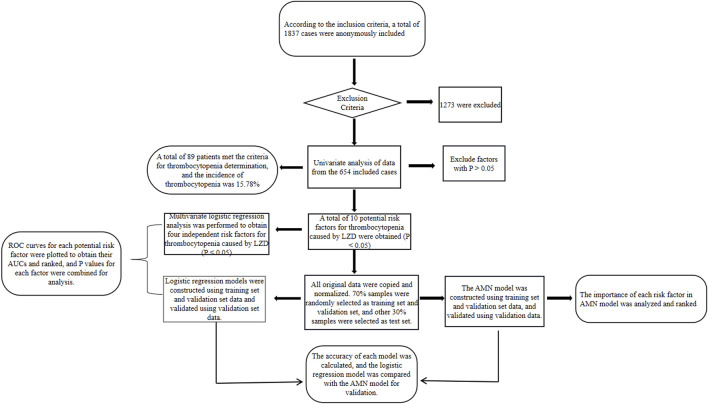
Flowchart of studies predicting risk factors for linezolid-induced thrombocytopenia.

In order to increase the rate of gradient descent, the data were normalized so that the processed data met the standard normal distribution; 70% of the samples were randomly selected as the training set along with the validation set for constructing the model, and the remaining 30% were used as the test set for validating the accuracy of the model. The study flow chart is shown in Figure 1.

## 3 Results

### 3.1 Case statistics

A total of 1,837 patients who had received linezolid treatment from 1 January 2011 to 1 January 2021 were collected, 1,273 patients who did not meet the requirements of the present study were excluded according to the exclusion criteria, and a total of 564 valid cases were finally included in the present study, including 357 males (63.30%) and 207 females (36.70%), with an average age of 50.26 ± 20.87 years, an age span of 1–100 years, average total days of medication of 6.90 ± 7.87 days, and a time span of use of 1–76 days. Of the 564 cases included, 89 were positive for meeting the criteria for thrombocytopenia in 2.2, which means that the incidence of linezolid-induced thrombocytopenia in the present study was 15.78%.

In addition, we also statistically analyzed the discharging department of the cases in the present study (Supplementary Table S1), with Geriatrics, Gastroenterology, and Urology department among the top three departments in the incidence of linezolid-induced thrombocytopenia, 40%, 35.71%, and 20.00%, respectively; of the 564 cases, 480 cases were from general departments, 84 cases were from intensive care units, and the incidence of thrombocytopenia in intensive care units was 35.71%, which was significantly higher than that in general departments (12.29%); in the intensive care units of each department, the incidence of thrombocytopenia in cardiovascular surgical care units was the highest, 63.64%.

### 3.2 Univariate analysis

The results of univariate analysis (Supplementary Table S2) showed that there were significant differences in age, history of hypertension, history of malignancy, baseline platelet values, TP, ALB, AST, DBIL, urea, and CCr between patients who developed thrombocytopenia and those who did not (*p* < 0.05).

### 3.3 Multivariate analysis

The OR, 95% CI and *p*-value of each factor were calculated by multivariate analysis using the factors with *p* < 0.05 in univariate analysis as independent variables and the occurrence or absence of thrombocytopenia as dependent variables. As shown in Supplementary Table S3, urea, baseline platelet value, age, and TP were independent risk factors for linezolid-induced thrombocytopenia in multivariate analysis (*p* < 0.05).

### 3.4 Construction of logistic regression model

On the basis of taking the factors with *p* < 0.05 in the univariate analysis as independent variables and the occurrence or absence of thrombocytopenia as dependent variables, after the steps of constructing the logistic regression model, logistic regression model and equation were obtained, and the logistic regression equation was as follows:

$$\begin{array}{c} \text{Logit}\left(P\right)=0.42936452\text{ XUrea}-0.72150774\text{ XPLT}+0.36437262\text{ XHTN}+0.42472864\text{ XAge}-0.11102075\text{ XCcr}+0.1818322\text{ XDBIL}\\ \;-0.06335601\text{ XALB}+0.13845654\text{ XAST}+0.20871324\text{ XMT}-0.28616011\text{ XTP}-2.19748157 \end{array}$$

Where: XUrea, XPLT, XHTN, XAge, XCcr, XDBIL, XALB, XAST, XMT, and XTP denote Urea, platelet baseline value, history of hypertension, age, Ccr, DBIL, ALB, AST, history of malignancy, and TP, respectively.

### 3.5 Construction of ANN model

**FIGURE 2:**
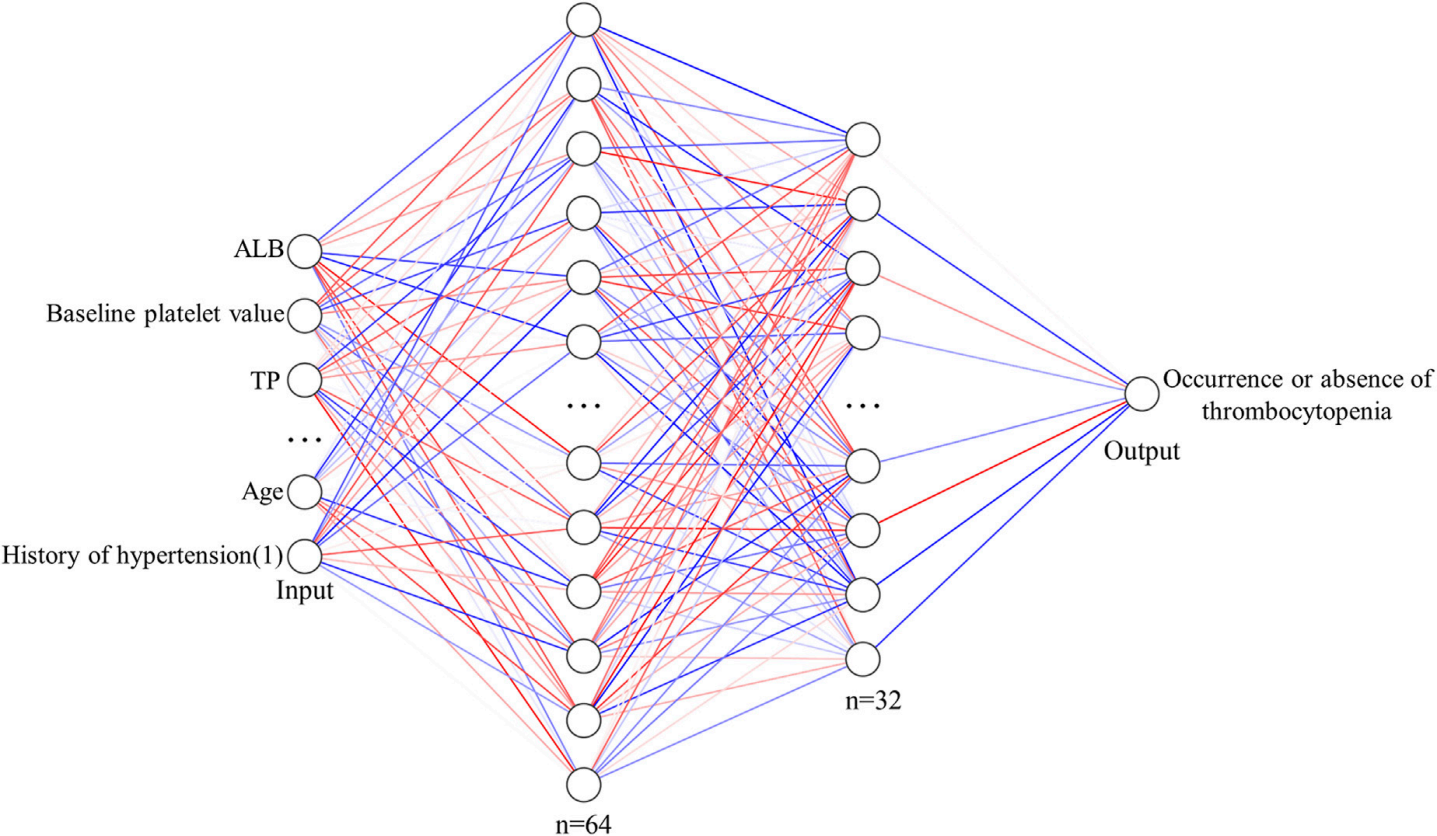
Structural diagram of ANN model for predicting the risk of thrombocytopenia caused by linezolid.

The ANN model is implemented on the PyTorch1.10 deep learning framework with Python3.8. Factors with *p* < 0.05 in univariate analysis were used as independent variables, and the occurrence or absence of thrombocytopenia was the dependent variable. As shown in Figure 2, ANN consists of two fully connected hidden layers connected sequentially, and the numbers of neurons in these layers are 64 and 32, respectively. The activation function of the hidden layer is ReLU and batch normalization is applied for the features output from the hidden layer. The Binary Cross Entropy is used as a loss function for training.

The data were preprocessed before being entered into the ANN. In detail, continuous factors were normalized by the max and min values of this factor. Discrete variables were coded by one-hot.

### 3.6 Validation of logistic regression model compared to ANN model

The obtained logistic regression model was subjected to the Hosmer-Lemeshow test, and the results are shown in Supplementary Table S4, resulting in a χ2 value of 12.493 and a *p*-value of 0.131 > 0.05, indicating that the logistic regression model constructed in the present study fitted well. Predictive validation of the logistic regression model with the test set yielded a prediction accuracy of 86.14%, with an AUC of 0.796. According to the prediction results of the logistic regression model, the sensitivity and specificity of each potential risk factor were calculated, and their AUC and ranking were obtained. The results are shown in Supplementary Table S5. In the present study, the factors with AUC >0.65 were considered to be well correlated, and then by combining again the *p* values of each potential risk factor in the multifactorial analysis, it can be concluded that urea, baseline platelet values, and age are significant risk factors for linezolid-induced thrombocytopenia as predicted by the logistic regression model.

**FIGURE 3:**
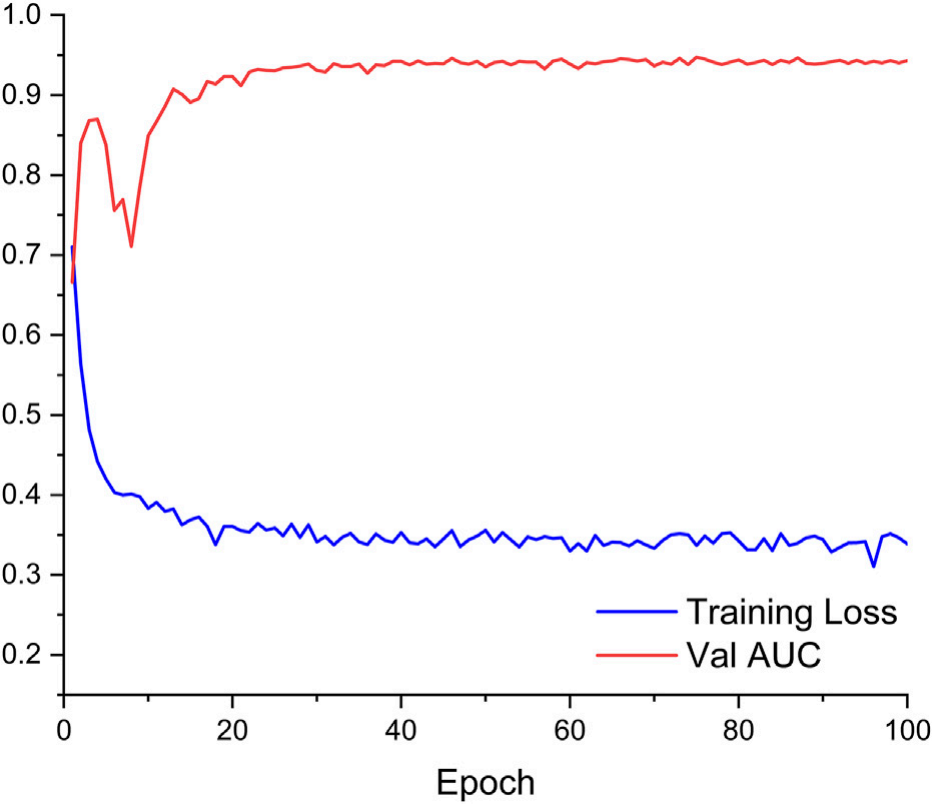
Training loss and AUC on validation dataset of ANN model during training process.

**FIGURE 4:**
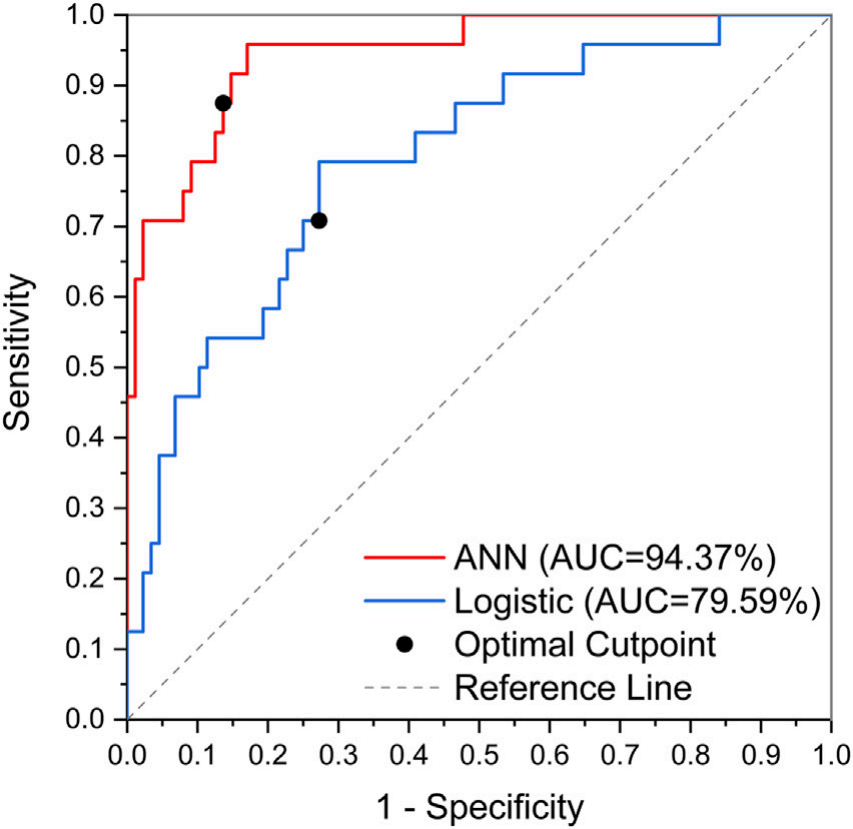
The ROC curves and AUCs of ANN and Logistic models on test dataset.

**FIGURE 5:**
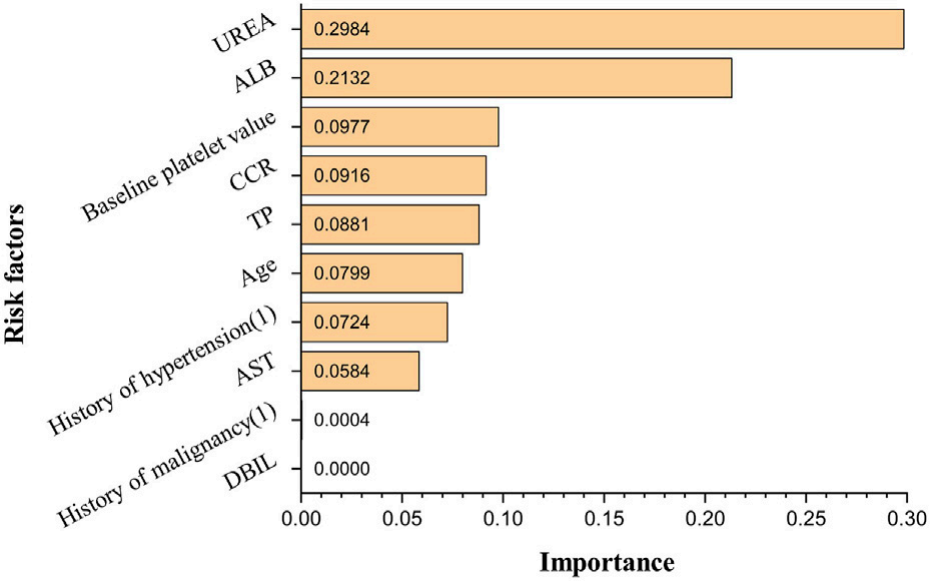
The importance and ranking diagram of potential risk factors in ANN model.

The obtained ANN model was validated for prediction using the test set with an accuracy of 96.32%, with an AUC of 0.944, indicating that the ANN model constructed in the present study has good predictive ability. Figure 3 shows the ROC curves and AUCs of ANN and Logistic models on the test dataset. We used SMOTE to augment the training data to alleviate overfitting and data imbalance. We set up batch-normal and drop-out layers after each layer of the ANN, which effectively avoids overfitting. As shown in Figure 4, we have plotted the loss and validation AUC curves of the ANN model during the training process. As can be seen from the curves, both the loss and the AUC on the validation set gradually remain stable with training, and there is no significant overfitting. This demonstrates the generalizability of the ANN we used. Figure 5 shows the importance and ranking of each potential risk factor obtained from the ANN model analysis, and urea, baseline platelet value, and ALB are among the top three important risk factors for ANN model prediction of linezolid-induced thrombocytopenia.

## 4. Discussion

Of the 564 cases included in the study, 89 developed thrombocytopenia, and the incidence of linezolid-induced thrombocytopenia was 15.78%; univariate analysis showed that age, history of hypertension, history of malignancy, baseline platelet value, TP, ALB, AST, DBIL, urea, and Ccr were potential risk factors for linezolid-induced thrombocytopenia (*p* < 0.05); in multivariate analysis, urea, baseline platelet value, age, and TP were independent risk factors for linezolid-induced thrombocytopenia (*p* < 0.05). According to the logistic regression model and in combination with the AUC of each potential risk factor and *p*-value in multivariate analysis, it is concluded that urea, baseline platelet value, and age were possible risk factors for linezolid-induced thrombocytopenia predicted by the logistic regression model. The importance of each potential risk factor obtained by ANN model analysis showed that urea, baseline platelet value, and ALB were among the top three possible risk factors for linezolid-induced thrombocytopenia predicted by the ANN model. Multiple studies have demonstrated a positive association between renal insufficiency and elevated concentrations of Linezolid, thereby increasing the risk of thrombocytopenia. Specifically, the risk of thrombocytopenia is found to be significantly elevated by 2-fold, 8-fold, and 9-fold in patients with mild, moderate, and severe renal insufficiency, respectively (Hanai et al., 2016b; Crass et al., 2019). Notably, Matsumoto K et al. (Matsumoto, 2012) have reported a negative correlation between Linezolid clearance and blood urea nitrogen levels. Similarly, Lin YH et al. (Lin et al., 2006) have observed a higher frequency of elevated blood urea nitrogen in patients with impaired renal function at the initiation of treatment, aligning with the findings of the present study. Urea, as the end product of protein metabolism, is subject to various influencing factors including intake, disease, and renal excretion capacity. In the current study, the thrombocytopenia group exhibited a higher mean age and a greater proportion of comorbidities such as hypertension, diabetes, and malignant tumors compared to the non-thrombocytopenia group. These factors may contribute to the observed elevation in blood urea nitrogen levels, suggesting that it may serve as a more reliable indicator of heightened catabolic state, increased renal burden, and an augmented risk of acute kidney injury. However, further investigations are warranted to validate these findings. It has been reported that patients with thrombocytopenia have significantly lower baseline platelet counts than those without thrombocytopenia, and patients with low baseline platelet counts should be closely monitored (Choi et al., 2019; Qin et al., 2022). Studies have shown that patients receiving human serum albumin therapy during Linezolid treatment have a low incidence of thrombocytopenia, and there is a significant negative correlation between serum albumin concentration and Linezolid-induced thrombocytopenia (Zhang et al., 2023). Chen et al. (Chen et al., 2012)found a high correlation between serum albumin concentration ≤33.5 g/L and thrombocytopenia, which is consistent with the results of our study with the mean albumin concentration of 33.48 g/L in the thrombocytopenia group. The decrease in albumin can reduce the protein binding rate of Linezolid in plasma and alter its distribution, leading to increased drug exposure and the occurrence of thrombocytopenia (Cattaneo et al., 2023). The findings need to be validated by further research as well. The results showed that the prediction accuracy of the logistic regression model was 86.14%, while that of the ANN model was 96.32%, which was much higher than that of the logistic regression model. Therefore, it can be concluded that the artificial neural network model constructed in the present study can be well predictive of the risk factors and their occurrence of linezolid-induced thrombocytopenia. In addition, in the actual clinical environment, there is always a complex nonlinear mixture of predictor variables. Most studies on linezolid-induced thrombocytopenia are mainly analyzed by retrospective analysis of small samples or traditional linear multivariate statistics, which can’t perform the analysis of large samples and complex relationships between multiple factors. Hence the combination of ANN with traditional statistical analysis (such as logistic regression analysis and log-likelihood analysis) could be a more effective solution to the problem of analyzing complex, multifactorial data in real-world data.

Although a risk prediction model for adverse drug reactions was successfully constructed using ANNs, ANNs also have some limitations compared with logistic regression analysis. First, ANNs have certain “Black Box” properties, that is, ANNs cannot explain why the constructed model can be obtained, nor can they give specific model equations. Therefore, the ANN model can only show the importance of each independent variable and rank them, but can not calculate their sensitivity and specificity, so their ROC curve and Youden index cannot be obtained to determine the cutoff value of each independent variable. This disadvantage will hinder its wider clinical use. Second, the ANN model requires high data diversity, and if the amount of data is too small, or the data source is not wide enough, the performance statistics (prediction accuracy, sensitivity, specificity, etc.) may become unstable and unreliable, meaning that false negative or false positive errors will have a greater impact on the analysis results, and will greatly affect the prediction accuracy of the ANN model. Third, ANNs have the risk of overtraining and the possibility of overfitting models, which may provide overconfidence predictions. Finally, in terms of clinical application, ANN requires the use of certain statistical analysis software or the possession of a programming language basis, so it is difficult to widely use the ANN model in clinical practice at present. However, Pergialiotis V et al. (Pergialiotis et al., 2018) believe that these problems can be solved by including more patients (in addition to the need for special statistical analysis software), that is, establishing a larger database, which is necessary to construct a more accurate and safe ANN model.

The present study also has some limitations. First, the study was a single-center study and there may have been bias in patient selection, which would reduce the likelihood of extrapolating the results to other populations, necessitating multi-center, bigger sample sizes in future research. Second, the number of samples remains small relative to possible risk factors, and there are more missing values for some variables (patient height, weight). The study drew on Johnsson J’s method (Johnsson et al., 2020) and replaced them with the mean of continuous variables. Third, only ROC curves of each potential risk factor were plotted in the logistic regression model, while Youden index was not further obtained to determine the cutoff value of each potential risk factor. Fourth, although heparin use may lower platelet counts during actual clinical treatment, the univariate analysis in this study did not reveal a significant difference in the impact of combining heparin use on the outcomes. This could be due to the study experiencing a small sample size, and more research is required to determine the impact of combining heparin use on platelets. In addition, for some other risk factors, including trough concentrations of linezolid, PaCO_2_, APACHE II, etc., the corresponding data could not be extracted due to conditional limitations, so their association with thrombocytopenia could not be assessed.

To the best of our knowledge, this is the first study to use the ANN model to predict risk factors for linezolid-induced thrombocytopenia from real-world medical data. In the present study, the ANN model built based on 10 indicators such as age, history of hypertension, history of malignant tumor, baseline platelet value, TP, ALB, AST, DBIL, Urea and Ccr could effectively predict the risk of linezolid-induced platelet reduction, with an accuracy of 96.32%, which was significantly higher than that of Logistic regression model. ANN model shows good performance in predicting adverse drug reactions, which can help clinicians and clinical pharmacists to predict adverse drug reactions more accurately, choose more appropriate drug treatment plans, and guide patients to individual drug use.

## Data Availability

The raw data supporting the conclusion of this article will be made available by the authors, without undue reservation.
