# Supplementary material for: Prediction of risk factors for linezolid-induced thrombocytopenia based on neural network model
Source: Front Pharmacol. 2024 Feb 21;15:1292828. doi: 10.3389/fphar.2024.1292828 (PMC10915059; doi:10.3389/fphar.2024.1292828)
Supplement: Supplementary file 1 [file Table1.DOCX]

**Supplement Tables**

Supplement Table 1 Statistics of discharging departments of 564 cases

| Discharging department  (General department) | Total number of cases | Number of positive cases | Discharging department  (Intensive care unit) | Total number of cases | Number of positive cases |
| --- | --- | --- | --- | --- | --- |
| Pediatrics | 2 |  | Pediatric Care Unit | 2 |  |
| Otolaryngology | 2 |  | Neonatal Care Unit | 2 |  |
| Hepatobiliary, Pancreatic and Splenic Surgery | 3 |  |  |  |  |
| Orthopedics | 21 | 1 |  |  |  |
| Respiratory Medicine | 2 |  | Respiratory Medicine Care Unit | 12 | 2 |
| Geriatrics | 55 | 22 |  |  |  |
| Physiotherapy | 2 |  |  |  |  |
| Urology | 20 | 4 |  |  |  |
| Burn & Skin Surgery | 73 | 10 |  |  |  |
| Neurology | 1 |  | Neurology Care Unit | 10 | 1 |
| Neurosurgery | 116 | 6 | Neurosurgical Care Unit | 21 | 4 |
| Nephrology | 3 |  |  |  |  |
| Gastroenterology | 10 | 3 | Gastroenterology Care Unit | 4 | 2 |
| Cardiovascular Surgery | 165 | 13 | Cardiovascular Surgery Care Unit | 33 | 21 |
| Transplant Center WARD | 3 |  |  |  |  |
| Plastic Surgery | 1 |  |  |  |  |
| Critical Care Medicine | 1 |  |  |  |  |
