# Supplementary material for: Prediction of risk factors for linezolid-induced thrombocytopenia based on neural network model
Source: Front Pharmacol. 2024 Feb 21;15:1292828. doi: 10.3389/fphar.2024.1292828 (PMC10915059; doi:10.3389/fphar.2024.1292828)
Supplement: Supplementary file 2 [file Table2.DOCX]

Supplement Table 2 Univariate analysis of risk factors for linezolid-induced thrombocytopenia

| Risk factors | Thrombocytopenia Group | Non-thrombocytopenia group | P |
| --- | --- | --- | --- |
|  | (n = 89) | (n = 475) |  |
| Gender (1)[n(%)] |  |  | 0.176^a^ |
| Male | 62(69.7%) | 295(62.1%) |  |
| Female | 27(30.3%) | 180(37.9%) |  |
| Age (‾x ± s, years) | 60.64±22.31 | 48.32±20.00 | <0.001^b^ |
| Height (‾x ± s, m) | 1.61±0.17 | 1.63±0.14 | 0.132^b^ |
| Body weight (‾x ± s, kg) | 65.96±13.36 | 64.40±14.77 | 0.353^b^ |
| BMI (‾x ± s, kg/m^2^) | 26.36±12.28 | 25.20±20.77 | 0.609^b^ |
| History of hypertension (1) [n(%)] |  |  | <0.001^a^ |
| Yes | 65(73.0%) | 202(42.5%) |  |
| No | 24(27.0%) | 273(57.5%) |  |
| History of diabetes (1) [n(%)] |  |  | 0.943^a^ |
| Yes | 26(29.2%) | 137(28.8%) |  |
| No | 63(70.8%) | 338(71.2%) |  |
| History of malignancy (1) [n(%)] |  |  | 0.021^a^ |
| Yes | 5(5.6%) | 7(1.5%) |  |
| No | 84(94.4%) | 468(98.5%) |  |
| Route of administration (1) [n(%)] |  |  | 0.066^a^ |
| Intravenous route | 76(85.4%) | 435(91.6%) |  |
| Non-intravenous route | 13(14.6%) | 40(8.4%) |  |
| Combined use of unfractionated heparin(1) [n(%)] |  |  | 0.07^a^ |
| Yes | 60(67.4%) | 271(57.1%) |  |
| No | 29(32.6%) | 204(42.9%) |  |
| Total days on drug (‾x ± s, days) | 6.38±9.18 | 7.00±7.60 | 0.550^b^ |
| Baseline platelet (‾x ± s, × 10^9/L) | 173.56±62.51 | 216.91±70.14 | <0.001^b^ |
| TP (‾x ± s, g/L) | 60.88±9.16 | 63.37±8.56 | 0.013^b^ |
| ALB (‾x ± s, g/L) | 33.48±4.51 | 35.44±5.27 | 0.001^b^ |
| ALT (‾x ± s, U/L) | 84.09±178.82 | 46.82±103.78 | 0.061^b^ |
| AST (‾x ± s, U/L) | 119.76±252.28 | 55.30±269.47 | 0.031^b^ |
| TBIL (‾x ± s, μmol/L) | 35.61±74.08 | 20.57±26.39 | 0.063^b^ |
| DBIL (‾x ± s, μmol/L) | 23.82±56.18 | 10.76±19.97 | 0.034^b^ |
| Urea (‾x ± s, mmol/L) | 16.20±12.50 | 9.03±7.01 | <0.001^b^ |
| Ccr (‾x ± s, ml/min) | 63.84±64.92 | 92.60±53.21 | <0.001^b^ |
