# Supplementary material for: Prediction of risk factors for linezolid-induced thrombocytopenia based on neural network model
Source: Front Pharmacol. 2024 Feb 21;15:1292828. doi: 10.3389/fphar.2024.1292828 (PMC10915059; doi:10.3389/fphar.2024.1292828)
Supplement: Supplementary file 3 [file Table5.DOCX]

Table 5 AUC and ranking of potential risk factors in logistic regression model

| Potential risk factor | AUC |
| --- | --- |
| Urea | 0.717 |
| Ccr | 0.712 |
| Baseline platelet | 0.689 |
| Age | 0.664 |
| History of hypertension (1) | 0.653 |
| DBIL | 0.618 |
| ALB | 0.616 |
| AST | 0.600 |
| TP | 0.598 |
| History of malignancy (1) | 0.521 |
