# Supplementary material for: Prediction of risk factors for linezolid-induced thrombocytopenia based on neural network model
Source: Front Pharmacol. 2024 Feb 21;15:1292828. doi: 10.3389/fphar.2024.1292828 (PMC10915059; doi:10.3389/fphar.2024.1292828)
Supplement: Supplementary file 4 [file Table3.DOCX]

Supplement Table 3 Multivariate analysis of risk factors for linezolid-induced thrombocytopenia

| Potential risk factor | B | S.E. | Wald | OR | 95% C.I. | | P |
| --- | --- | --- | --- | --- | --- | --- | --- |
|  |  |  |  |  | Lower limit | Upper limit |  |
| Age | 0.025 | 0.008 | 9.613 | 1.025 | 1.009 | 1.042 | 0.002 |
| History of hypertension (1) | -0.549 | 0.295 | 3.466 | 0.578 | 0.324 | 1.029 | 0.063 |
| History of malignancy (1) | -1.107 | 0.676 | 2.679 | 0.331 | 0.088 | 1.244 | 0.102 |
| Baseline platelet | -0.011 | 0.002 | 25.898 | 0.989 | 0.984 | 0.993 | 0.000 |
| TP | -0.048 | 0.024 | 4.103 | 0.953 | 0.909 | 0.998 | 0.043 |
| ALB | -0.002 | 0.039 | 0.002 | 0.998 | 0.924 | 1.078 | 0.966 |
| AST | 0.001 | 0.000 | 2.240 | 1.001 | 1.000 | 1.001 | 0.135 |
| DBIL | 0.006 | 0.004 | 2.499 | 1.006 | 0.999 | 1.014 | 0.114 |
| Urea | 0.073 | 0.017 | 18.833 | 1.076 | 1.041 | 1.112 | 0.000 |
| Ccr | 0.002 | 0.003 | 0.542 | 1.002 | 0.996 | 1.009 | 0.462 |
