# Supplementary material for: Prediction of risk factors for linezolid-induced thrombocytopenia based on neural network model
Source: Front Pharmacol. 2024 Feb 21;15:1292828. doi: 10.3389/fphar.2024.1292828 (PMC10915059; doi:10.3389/fphar.2024.1292828)
Supplement: Supplementary file 5 [file Table4.DOCX]

Supplement Table 4. Hosmer-Lemeshow test of logistic regression model

| χ^2^ | Degree of freedom | P |
| --- | --- | --- |
| 12.493 | 8 | 0.131 |
